# Repair of chronic and large rotator cuff tears using extra‐synovial autografts: An experimental study in rabbits

**DOI:** 10.1002/jeo2.12010

**Published:** 2024-02-28

**Authors:** Iosafat Pinto, Lazaros Kostretzis, Konstantinos Katakalos, George Kazakos, Angeliki Cheva, Athanasios Chatzisotiriou, Pericles Papadopoulos, Konstantinos Ditsios

**Affiliations:** ^1^ 2nd Orthopaedic Department, Aristotle University of Thessaloniki General Hospital of Thessaloniki “G.Gennimatas” Thessaloniki Greece; ^2^ Laboratory for Strength of Materials and Structures, Civil Engineering Department Aristotle University of Thessaloniki Thessaloniki Greece; ^3^ School of Veterinary Medicine Aristotle University of Thessaloniki Thessaloniki Greece; ^4^ Pathology Department, School of Medicine Aristotle University of Thessaloniki Thessaloniki Greece; ^5^ Physiology Department, School of Medicine of Aristotle University of Thessaloniki Thessaloniki Greece

**Keywords:** extra‐synovial autografts, in vivo, rabbit model, rotator cuff tear

## Abstract

**Purpose:**

To investigate whether and how extra‐synovial autografts can enhance the reconstruction of chronic and large rotator cuff tears in a rabbit subscapularis model.

**Methods:**

Twenty rabbits were used to create a large subscapularis tear bilaterally. Six weeks later, the right shoulder of each rabbit was operated to repair the tear with an extra‐synovial autograft, whereas the left shoulder did not undergo any surgery. At 6 and 12 weeks after the second procedure, the specimens underwent biomechanical and histological evaluation. Six more rabbits were used only as a normal reference.

**Results:**

Biomechanical evaluation demonstrated that the ultimate load to failure of the Graft group (184.1 ± 35.7 N) was significantly higher (*p* = 0.04) than that of the Defect group (144.5 ± 32.2 N) at 12 weeks after repair, rising to 76% of the normal subscapularis tendon tensile strength. Histological analysis revealed an enhanced healing environment with neoangiogenesis and decreased inflammatory response at the repair site. Moreover, the tendon maturing score of the Graft group increased substantially from 6 (15.8 ± 0.9) to 12 (23.1 ± 0.6) weeks after repair (*p* = 0.01).

**Conclusion:**

In vivo data support the efficacy of extra‐synovial autograft interposition in repairing chronic and large rotator cuff tears in a rabbit subscapularis model. The autografts were capable of enhancing the biomechanical properties of the repaired tendons, as evidenced by increased tensile strength, and forming new connective tissue simulating a fibrocartilage zone, as revealed by histological evaluation.

**Level of Evidence:**

N/A.

**Abbreviations**
ECM: extra‐cellular matrix patches
H&E: haematoxylin‐eosin
Maxload: ultimate load‐to‐failure
RC: rotator cuff
SSC: subscapularis

## BACKGROUND

Worldwide, shoulder pathology is the third most common musculoskeletal presentation in primary care [22]. Notably, rotator cuff (RC) disease is the most common cause of shoulder pain [6] and surgical intervention is often required. Despite advances in techniques, materials and operative options, the failure rates after repair remain high and range from 20% to 90% [10]. A large number of prognostic factors have been associated with increased risk of failure, but based on recent data re‐tear risk is mainly affected by older age and larger tears size [24].

Experimental studies on animal models have shown that an increase in repair tension is related with detrimental changes to the healing insertion site and that the tension required grows with time following detachment [12]. Therefore, in order to optimize healing in chronic and large RC tears we need to decrease the tension of repair. The utilization of grafts is a promising way to augment rotator cuff reconstruction sites, especially when dealing with large and chronic tears. Until recently, little research has been conducted on the usage of autografts in RC tears. Previous histological studies have shown that the vascular supply to RC tendons is not typical of intrasynovial or extra‐synovial tendons [4]. Extra‐synovial autografts were chosen to reconstruct RC tears, based on the superior mechanical properties they demonstrate compared to intrasynovial tendons, especially regarding the tensile strength [25].

More than 30 different animal species have been used to study RC tears [9]. Grumet et al. [13] introduced a model in which the subscapularis (SSC) in rabbits is used to study the supraspinatus in humans [20]. This model is based on the observation that the rabbit's supraspinatus, due to the relatively rudimentary nature of the acromion, does not traverse beneath it. Conversely, the subscapularis in rabbits travels under a bony arch, due to an additional bony prominence on the anterior aspect of the joint, contributing to the creation of a three‐sided bony tunnel. This unique relationship of the rabbit subscapularis tendon and the scapular bone tunnel, creates a condition similar to what is found in the human RC and the supraspinatus travelling under the acromion.

Our aim was to investigate whether extra‐synovial autografts can augment the reconstruction of chronic and large rotator cuff tears in a rabbit subscapularis model and the mechanisms thereof. We hypothesized that the autograft interposition could enhance the biomechanical performance and improve the healing process of the subscapularis tendon.

## METHODS

This study was conducted in accordance with the Animal Research: Reporting of In Vivo Experiments (ARRIVE) guidelines. All animal procedures were approved by our Institutional Bioethics Committee (Approval Number: 4108) and from the Regional Animal Health Department (Approval Number: 449339‐2585). All animals were housed individually at 22°C in their cages with free access to food and water. After surgery, all animals were allowed normal cage activity without immobilization.

### Study groups

**Figure 1:**
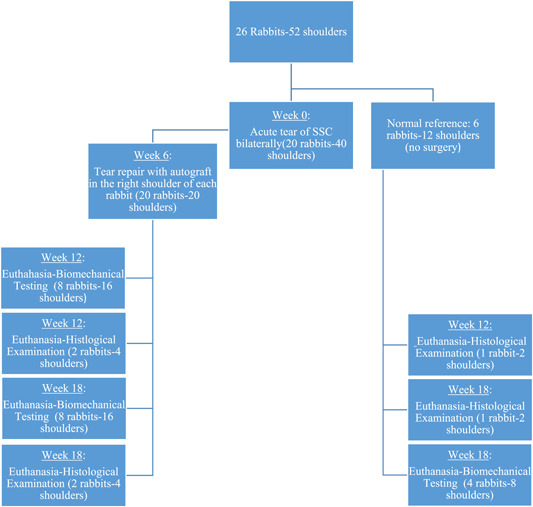
Overview of the study design and time course.

This study was designed so that the right shoulder of each rabbit can be compared with its left shoulder, thus creating one graft and one defect group respectively. Twenty‐six male New‐Zealand white rabbits (average age, 3 months; weight, 3 kg) were used in this study. Twenty of them were used for creating large and chronic RC tears, whereas the remaining six were used only as a normal reference. The study was divided into a two‐phase surgical procedure. Initially, a large U‐shaped tear was created in all rabbits (*n* = 20) bilaterally, thus the number of subscapularis tendons in total was twofold (*n* = 40). Six weeks later, the right shoulder of each rabbit was operated to repair the tear with the usage of extra‐synovial autograft, harvested at the same time from the ipsilateral hind paw (Graft Group). During the second operation, the left shoulder in all rabbits did not undergo any repair in order to assess the ability to self‐heal (Defect Group). At 6 and 12 weeks after the second procedure, eight randomly selected rabbits were euthanized and bilateral shoulders were harvested and used for biomechanical testing, respectively. At each time‐point, two more rabbits were euthanized and evaluated histologically (Figure 1).

### Tear creation

**Figure 2:**
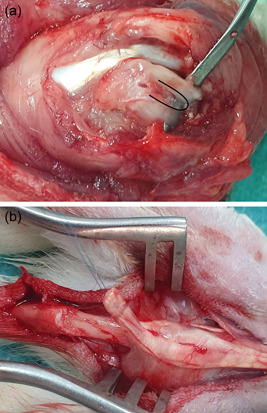
(a) Full thickness defect creation in subscapularis tendon (U shaped). (b) Harvest of the central slip of the extensor expansion complex from the second toe of the hind paw.

Rabbits were sedated by a qualified veterinary anesthetist with dexmedetomidine 80 μg/kg IM and tramadol 2 mg/kg IM, anesthetized using propofol and then intubated and maintained with isoflurane. Postoperative protocol included meloxicam 1 mg/kg SC (3 days) for analgesia, and 150 mg cefamandole IM (3 days) for antimicrobial prophylaxis. Two orthopaedic surgeons (I. P. and L. K.) performed all operations under loupe magnification. Both shoulders were shaved, prepped, and draped in the normal sterile fashion. A 3 cm longitudinal anterolateral skin incision was made on right and left shoulder consecutively. The Deltoid was retracted laterally, the pectoralis major was incised medial to its insertion and the subscapularis tendon was identified. Then, a full‐thickness defect was created by sharp dissection in the middle part of the tendon, extending from the footprint toward the myotendinous junction (Figure 2). This tear had a width of 50% of the tendon (superior–inferior direction) and a length of approximately 10 mm (mediolateral direction), simulating a large tear [5, 21]. The wound was then closed in layers.

### Tear repair

**Figure 3:**
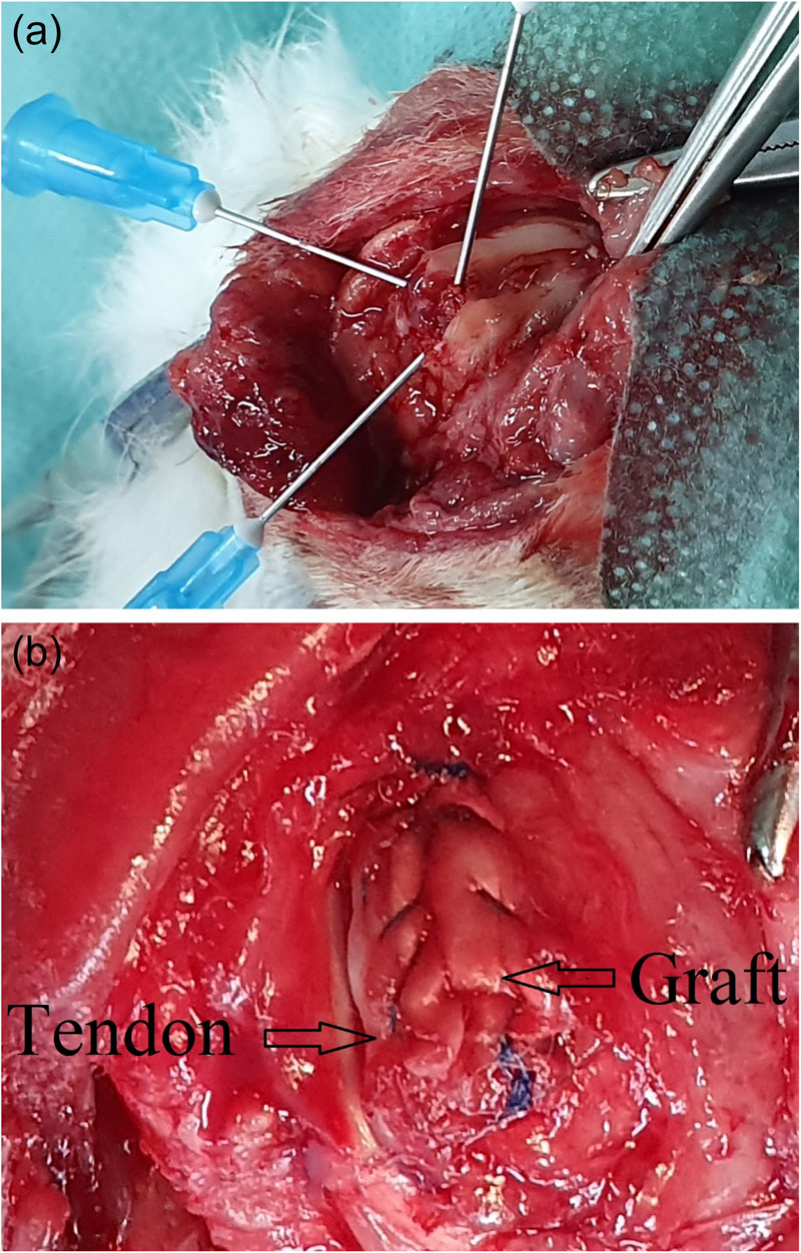
(a) Footprint preparation. The lesser tuberosity is debrided and two bone holes are drilled from the footprint to proximal metaphysis. (b) Reconstruction of subscapularis tendon defect with an extra‐synovial graft which is compressed to the footprint with transosseous technique and then sutured to the remaining SSC tendon.

After 6 weeks, under the same anesthesia, the second surgery was performed using the same approach for the right shoulder and a 3 cm longitudinal dorsal approach over the second toe of the hind paw for the graft. The central slip of the extensor expansion complex was harvested along with its paratenon (Figure 2), and then used to fully cover the defect of the subscapularis tendon. The footprint onto the lesser tuberosity was decorticated until bleeding occurred and two bone holes (diameter 0.8 mm) were drilled from the footprint to the proximal humeral metaphysis. We attached and secured with a Prolene 5‐0 suture one side of the graft to the footprint through the drill holes, and subsequently sutured the remaining graft to the surrounding subscapularis tendon (Figure 3). Both wounds were then closed in layers. After each operation, rabbits were daily monitored for signs of infection.

At the pre‐established time points, rabbits were euthanized with excessive IV dose of propofol. The entire scapula with all RC muscles and tendons along with the proximal humerus of each rabbit was harvested bilaterally. All specimens were gross examined for signs of infection, fibrosis and integrity.

### Biomechanical evaluation

**Figure 4:**
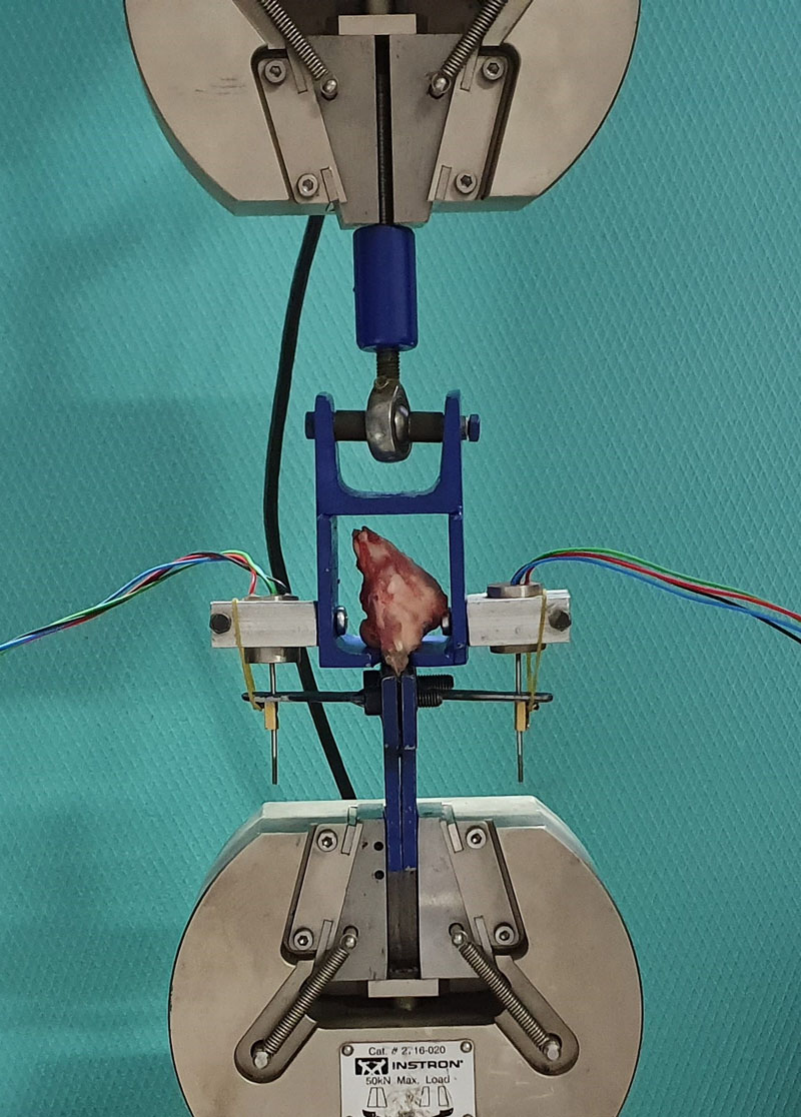
Biomechanical testing setup.

The total number of rabbits tested biomechanically was 20 (40 shoulders). Eight rabbits (16 shoulders) were tested at 6 weeks, and another eight (16 shoulders) at 12 weeks after repair along with four normal reference rabbits (8 shoulders). All specimens were wrapped in gauze, hydrated with saline solution and stored at 4°C for no more than 2 hours, until the biomechanical testing. After been thawed at room temperature, the scapula and the RC muscles were carefully dissected and removed, leaving only the subscapularis tendon and the humeral head attached. The SSC tendon was sutured with a 2‐0 Ethibond (J & J Medical N.V.) suture in a Krakow style, 5 mm proximal to its insertion to the humeral head. The free end of the tendon was then fixed in a holding clamp with grit sandpaper and cyanoacrylate adhesive to reduce slippage between the subscapularis tendon and the holding clamp during longitudinal stressing. For the purposes of this study we designed and manufactured a custom‐made clamping system for the humeral head, which secures the head and also permits the SSC tendon to pass through a hole. Care was taken to ensure symmetric tension across the tendon before clamping (Figure 4). A materials testing machine (Instron 5969) was used for mechanical evaluation of the specimens in combination with a Linear Variable Differential Transformer (LVDT) system (Micro‐measurement) to evaluate more accurately the tendon's strain. All data collected was recorded with the use of Data Acquisition System (DAS, Kyowa).

After a preload of 15 N was applied for 30 sec for preconditioning, the specimen was then subjected to a loading rate of 1 mm/s until failure. A stress‐strain curve was obtained from the load and displacement data. The ultimate tensile load at which the tendon failed, elongation, stiffness and mode of failure during tensile testing were also recorded.

### Histological evaluation

The total number of rabbits evaluated histologically was 6 (12 shoulders). Two rabbits (4 shoulders) were evaluated at 6 weeks, and another two (4 shoulders) at 12 weeks after repair. Another two matching in age rabbits (4 shoulders), that did not undergo any procedure, were used as normal reference at 6 (1 rabbit) and 12 (1 rabbit) weeks after repair. Initially, the complexes of the subscapularis tendon and the humeral head were fixed in 10% neutralized formalin for 24 h and then decalcified in 6.5% nitric acid (Pan Reac Applichem) for 4 h. After decalcification, tissues were embedded in paraffin and longitudinal sections of 3 μm in thickness were obtained from the tendon to bone region. Finally, tissue sections were stained with haematoxylin‐eosin (H&E) and Masson trichrome. All sections were evaluated by a histopathologist, who was blinded regarding the group of the specimen, using a stereoscopic microscope (Nikon Eclipse E600) with an integrated camera system (Nikon model DS‐Fi2‐L2). Specimens were evaluated for collagen fiber organization, cellularity, vascularization and the presence of inflammatory response. Moreover, the Modified Tendon Maturing Score by Ide et al. [15] was utilized to quantitatively evaluate the extra‐synovial graft regarding cellularity, tenocytes, cells orientation, vascularity, fiber diameter, fiber orientation and remodelling at the insertion site. This scoring system has a maximum score of 28 points.

### Power and statistical analysis

Ultimate load‐to‐failure (Maxload) in the biomechanical testing was chosen as the primary outcome of this study. Power analysis using the R Project 4.2.3 software (R core team) indicated that a sample size of 8 was required for biomechanical studies to detect a significant difference in ultimate load‐to‐failure between the Graft and the Defect group in each time‐point (mean difference, 55 N; standard deviation, 35 N; a‐error, 0.05; b‐error, 0.2), based on values from a previous study [21].

The statistical analysis was conducted using Stata 16 software (StataCorp). The pooled *t* test was used to determine the statistical difference in ultimate load‐to‐failure, stiffness and elongation between the right and left shoulders and the Shapiro–Wilk test was used to evaluate the sample data for normal distribution. All data were presented as mean ± standard deviation and in all cases the significance level of *p* < 0.05 was used.

## RESULTS

### Animal surgery

All rabbits survived the surgical procedure, and there were no instances of infection or other postoperative complications. Two rabbits chewed their sutures and required reclosure of the surgical wounds.

### Biomechanical evaluation

**Figure 5:**
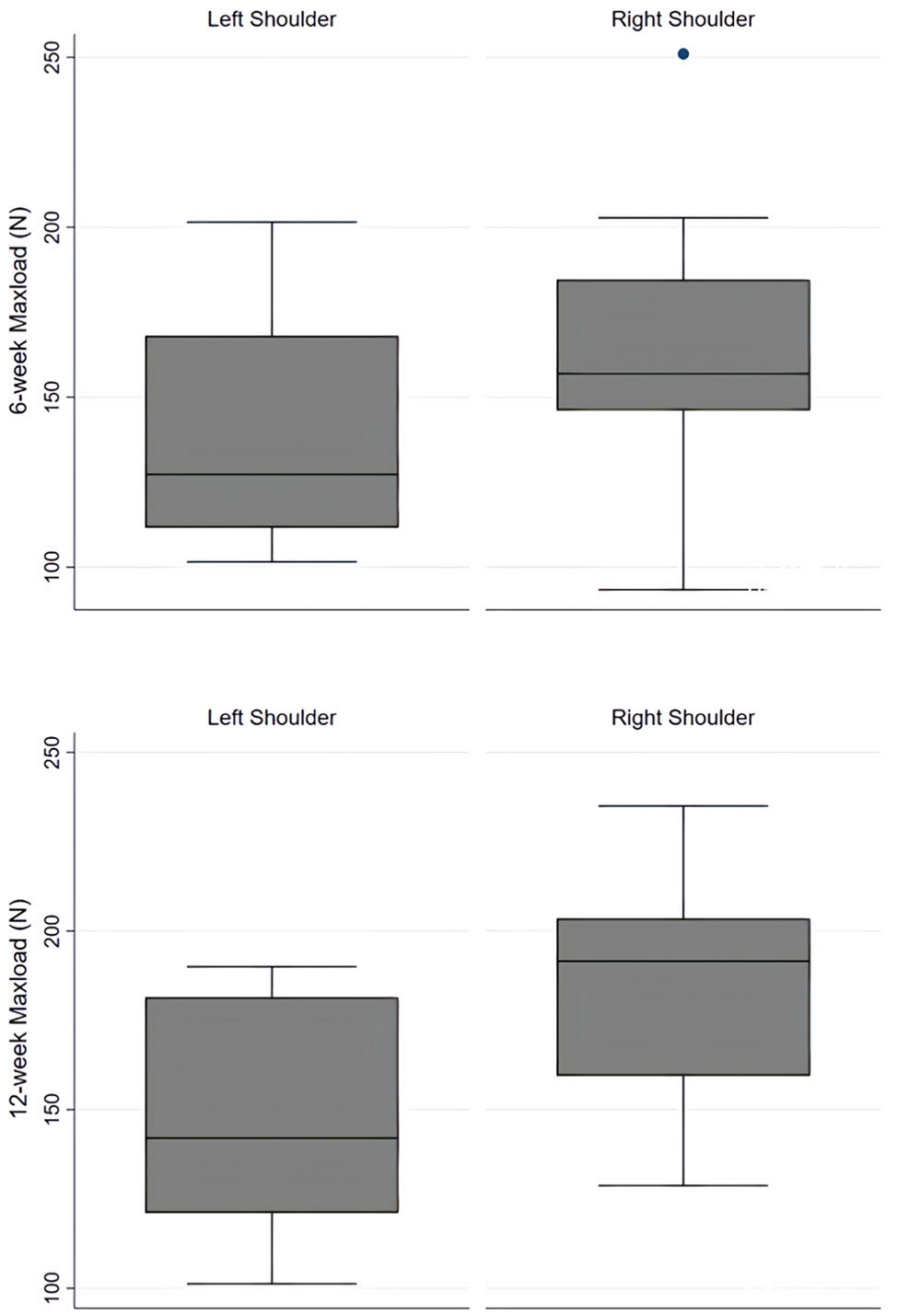
Vertical box plots of ultimate load‐to‐failure (Maxload) at 6 and 12 weeks after repair. The centred line represents the median, the top and bottom edges of the coloured area indicate the 25th and 75th percentile values, whereas the two extreme horizontal lines are the lower and upper adjacent values lying 1.5 iqr (interquartile range) away from the 25th and 75th percentiles. Dots are plotted beyond the 1.5 iqr to denote outlier values of each variable's distribution.

**Figure 6:**
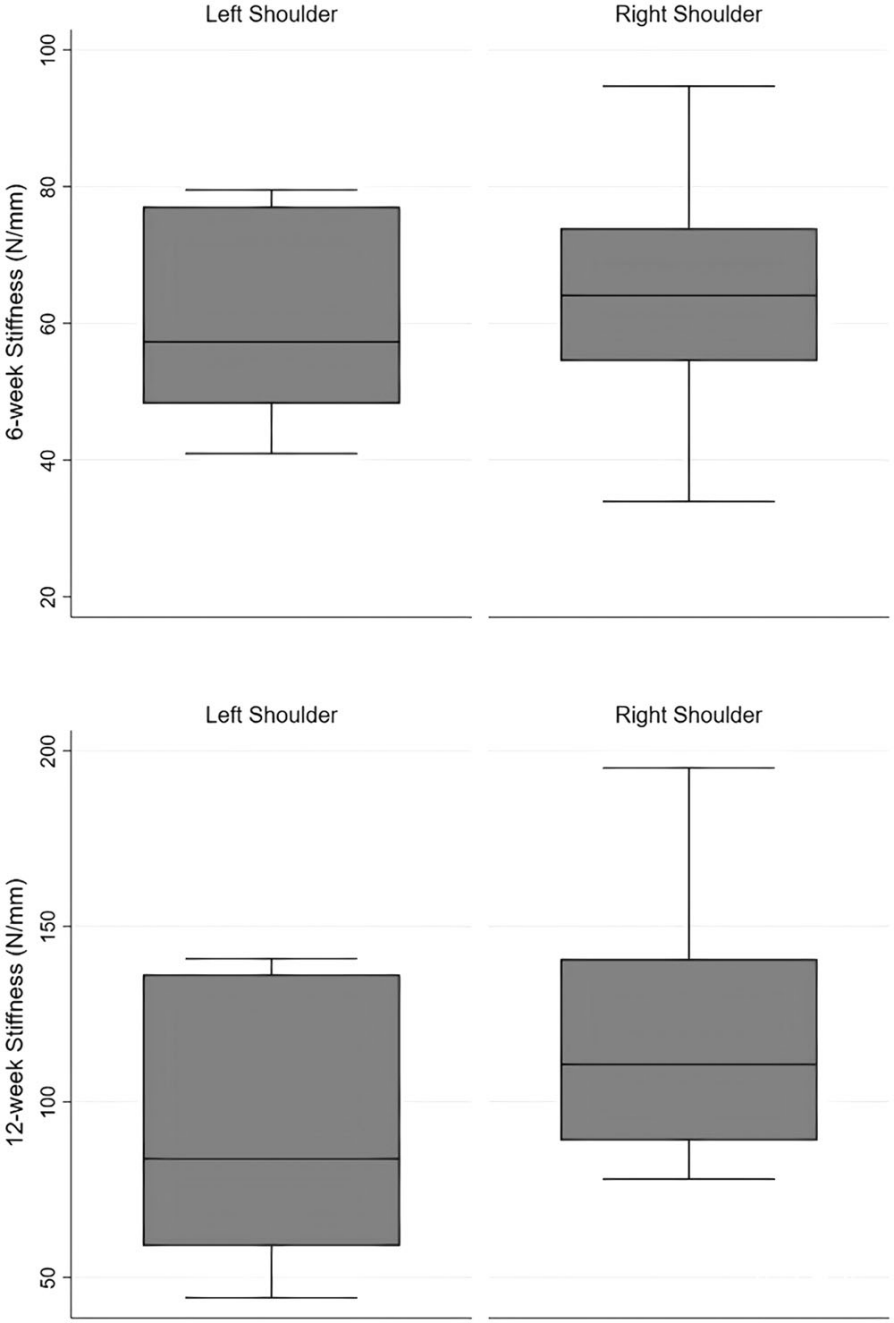
Vertical box plots of stiffness at 6 and 12 weeks after repair. The centred line represents the median, the top and bottom edges of the coloured area indicate the 25th and 75th percentile values, whereas the two extreme horizontal lines are the lower and upper adjacent values lying 1.5 interquartile range (iqr) away from the 25th and 75th percentiles. Dots are plotted beyond the 1.5 iqr to denote outlier values of each variable's distribution.

**Figure 7:**
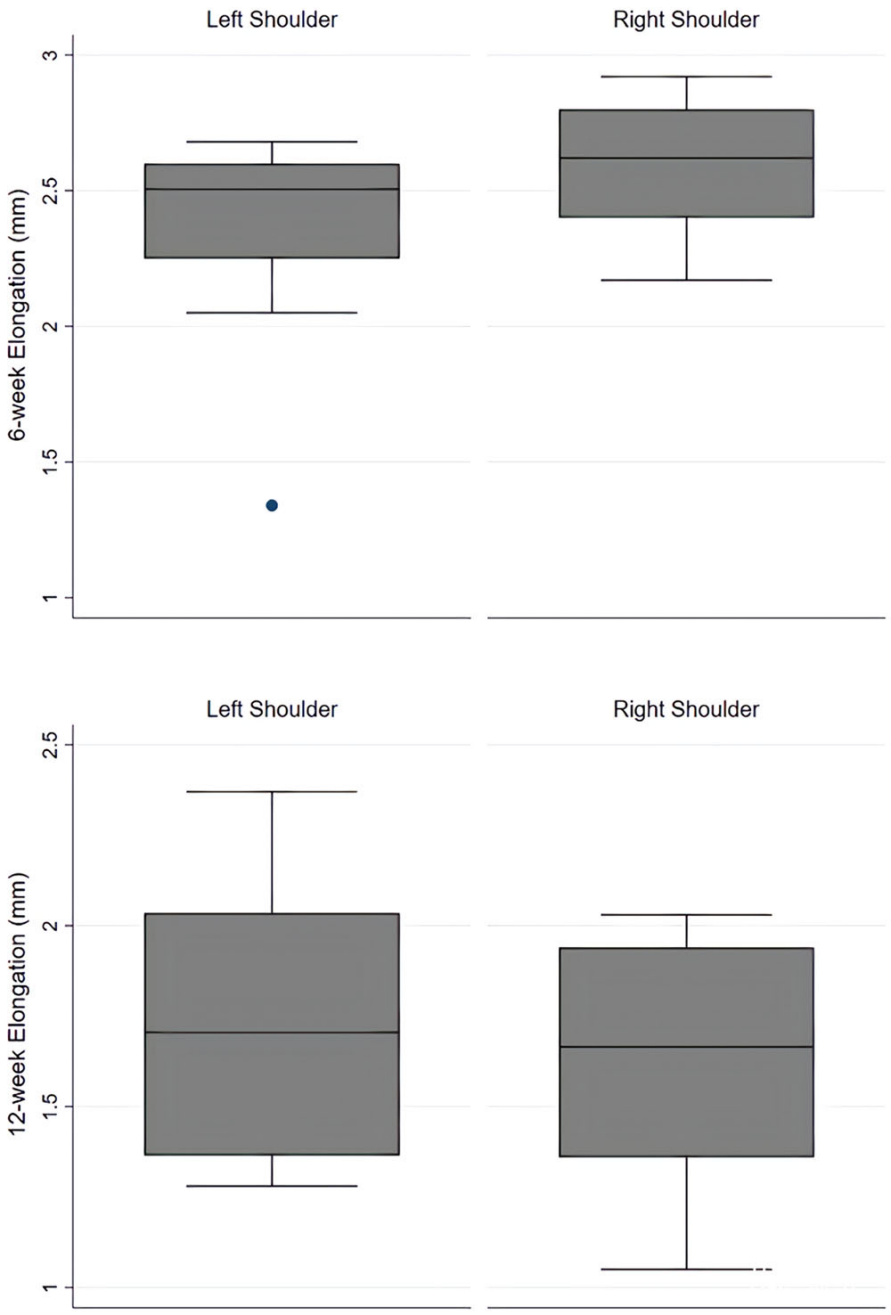
Vertical box plots of elongation at 6 and 12 weeks after repair. The centred line represents the median, the top and bottom edges of the coloured area indicate the 25th and 75th percentile values, whereas the two extreme horizontal lines are the lower and upper adjacent values lying 1.5 interquartile range (iqr) away from the 25th and 75th percentiles. Dots are plotted beyond the 1.5 iqr to denote outlier values of each variable's distribution.

Results of biomechanical evaluation for the two groups tested are shown in Table 1. The difference of mean ultimate load‐to‐failure between right (164.9 ± 45.2 N) and left (139.6 ± 37.6 N) shoulders at 6 weeks after repair was not statistically significant. On the contrary, at 12 weeks after repair mean ultimate load‐to‐failure values were significantly higher in right (184.1 ± 35.7 N) than in left (144.5 ± 32.2 N) shoulders (*p* = 0.04) (Figure 5). In addition, the normal reference shoulders demonstrated significantly higher ultimate load‐to‐failure values (243.1 ± 23.89 N) compared to both Graft (76% of normal SSC) and Defect group (59% of normal SSC) at 12 weeks after repair (*p* = 0.001). Moreover, right shoulders demonstrated higher stiffness at 12 weeks after repair compared to left shoulders, but this was weakly statistically significant (Figure 6). No significant differences were found between right and left shoulders for elongation at any time‐point (Figure 7).

**Table 1:** Summary statistics.

	Specimens	Mean R	Mean L	*p* Value
Max Load R vs. Max Load L, 6 weeks	8	164.9 ± 45.2	139.6 ± 37.6	0.23
Elongation R vs. Elongation L, 6 weeks	8	2.59 ± 0.26	2.34 ± 0.45	0.19
Stiffness R vs. Stiffness L, 6 weeks	8	64.2 ± 17.41	60.7 ± 14.38	0.72
Max Load R vs. Max Load L, 12 weeks	8	184.1 ± 35.7	144.5 ± 33.2	0.04
Elongation R vs. Elongation L, 12 weeks	8	1.63 ± 0.36	1.77 ± 0.37	0.45
Stiffness R vs. Stiffness L, 12 weeks	8	119 ± 39.25	90 ± 37.14	0.15

Three modes of failure were observed during biomechanical testing: (1) At the insertion (tendon‐bone junction) (2) At the midsubstance (middle part of the tendon), and (3) At the clamp (proximal part of the tendon). Failure at the midsubstance of tendons suggests strong tendon‐to‐bone healing, whereas rupture at the tendon‐bone junctions suggests relatively weak tendon‐to‐bone healing [27]. At 6 weeks, failure through midsubstance occurred evenly for right and left shoulders, representing 50% of the specimens. Two specimens from the defect group and three specimens from the graft group failed at the insertion. Failure at the clamp was seen two times at the defect group and one time at the graft group. At 12 weeks, midsubstance failure occurred seven (87.5%) and five (62.5%) times in right and left shoulders, whereas failure at the insertion was seen one (12.5%) and three (37.5%) times respectively. None failed at the clamp.

### Histological evaluation

**Figure 8:**
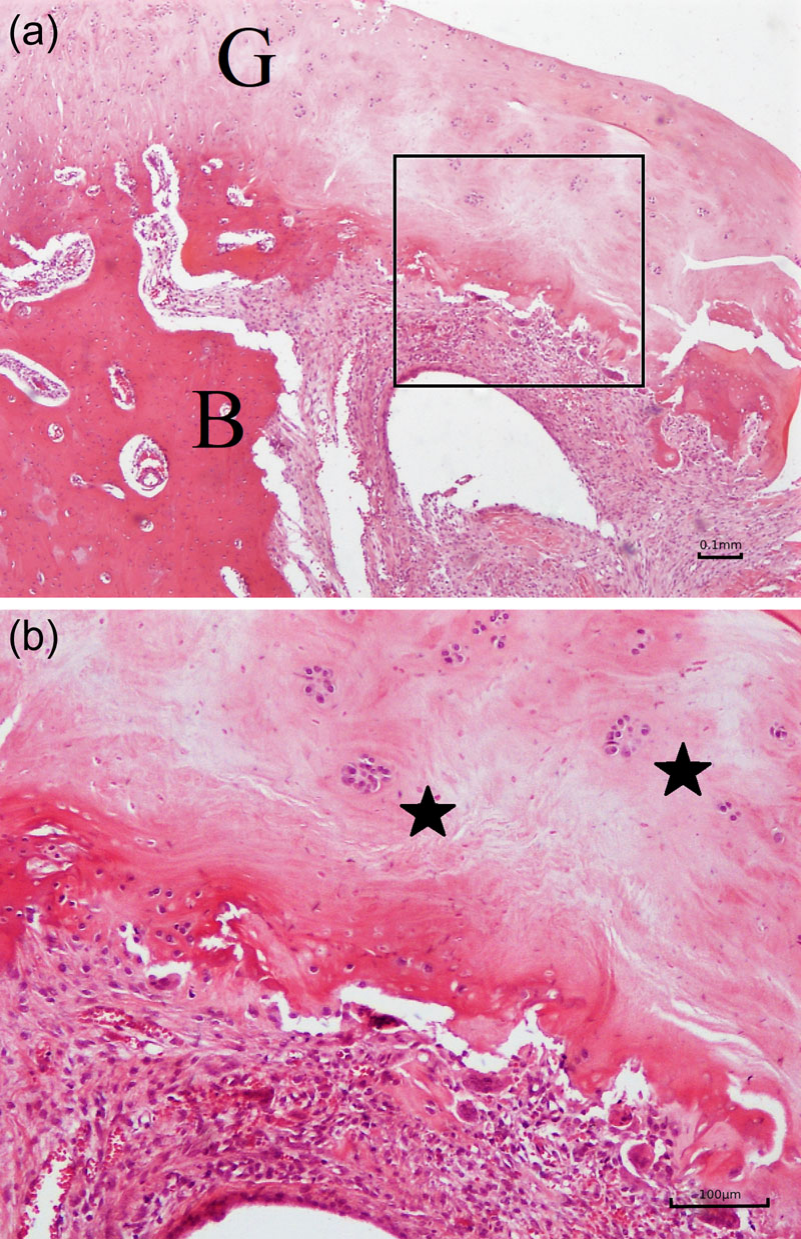
Images of the graft group at the repair site 6 weeks after repair. (a) Haematoxylin‐eosin staining ×40 (*B* bone of humeral head, *G* extrasynovial tendon graft). (b) Haematoxylin‐eosin staining ×100 with multinucleated giant cells (*pentagram*) and absence of contact between the graft and the bone.

**Figure 9:**
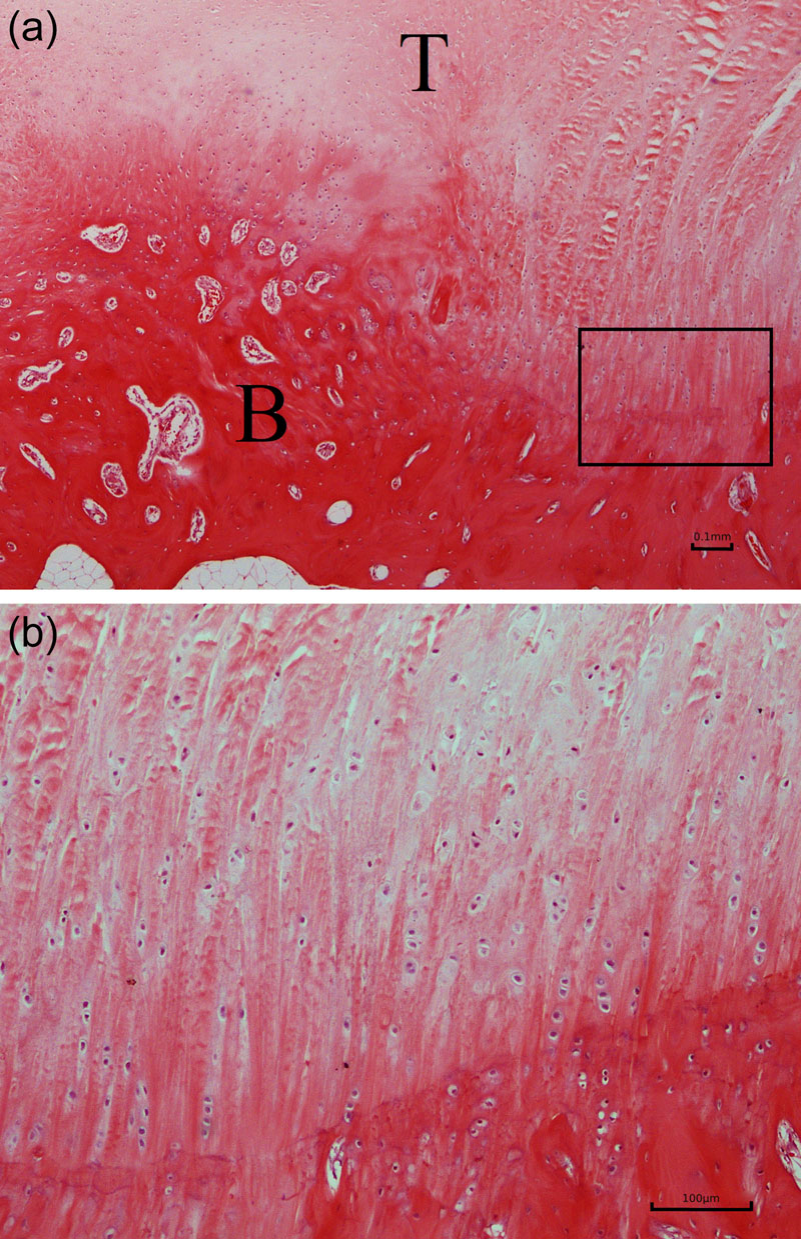
Images of the defect group at the repair site. (a) Haematoxylin‐eosin staining ×40 (*B* bone of humeral head, *T* original tendon stamp). (b) Haematoxylin eosin‐staining ×100.

There were no signs of infection at the surgical site in any of the specimens examined, and all grafts were found undamaged. At 6 weeks after repair, newly formed connective tissue was seen at the tendon to bone interface. However, this tissue was characterized by chondrocyte disorganization, and the collagen fibers were not yet fully anchored to the bone. The distal stump of the graft showed new vessel formation and abundant inflammatory cells at the repair site (Figure 8). In the defect group, the void was still macroscopically obvious and we verified the low volume of self‐healing capacity in rotator cuff tears. Microscopically, in the remaining original tendon stump we did not identify any significant healing activity (Figure 9).

**Figure 10:**
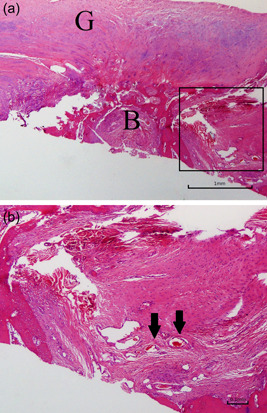
Images of the graft group at the repair site 12 weeks after repair. (a) Haematoxylin‐eosin staining ×20 (*B* bone of humeral head, *G* extrasynovial tendon graft). (b) Haematoxylin‐eosin staining ×40 with newly formed blood vessels (*arrowhead*).

**Figure 11:**
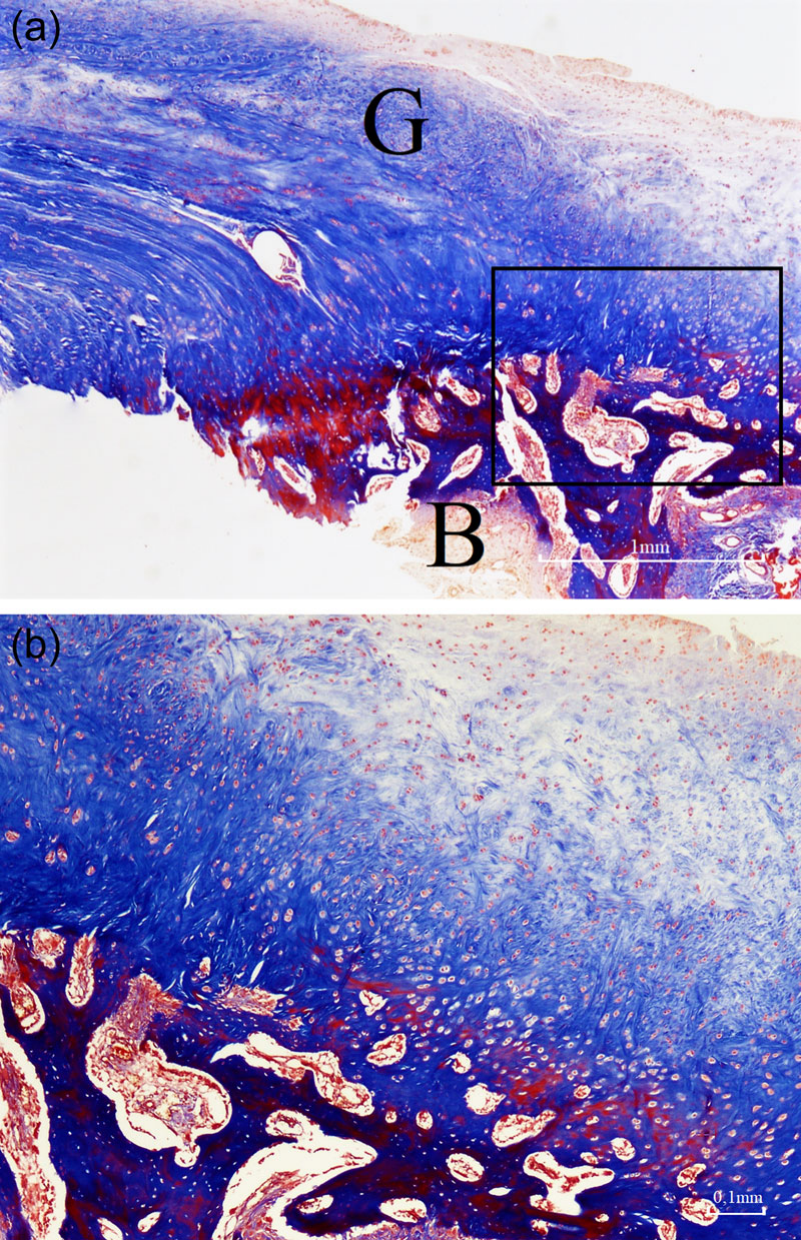
Images of the graft group at the repair site 12 weeks after repair. (a) Masson's trichrome staining ×20 (*B* bone of humeral head, *G* extrasynovial tendon graft). (b) Masson's trichrome staining ×40 where collagen fibers have fully anchored to the bone.

At 12 weeks after repair, histological examination revealed evidence of neoangiogenesis and decreased inflammatory response at the repair site. Upon microscopy, the newly formed connective tissue appeared denser with parallel collagen fibers, simulating a fibrocartilage zone (Figure 10). In addition, Masson's trichrome stain highlighted that the collagen fiber continuity between the graft and the bone was complete (Figure 11). In the defect group, fibrocartilage was absent and no inflammatory response was noted either.

Histological analysis also revealed that the Modified Tendon Maturing Score of the Graft group increased substantially from 6 (15.8 ± 0.9) to 12 (23.1 ± 0.6) weeks after repair (*p* = 0.01).

## DISCUSSION

This study investigated the feasibility of managing chronic and large RC tears with extra‐synovial autografts in a rabbit SSC model. A bridging technique was implemented to fill the defect of the subscapularis without tensioning the retracted tendon and without altering the length of the degenerated muscle. As a result, the tears were repaired, and the contact area of the tendon enthesis was increased with the minimum tension required.

Despite satisfactory results having been demonstrated with RC repair, a high incidence of such tears never heal [8]. Most of those repairs fail within the first 3–6 months due to tissue pulling through the sutures [18]. Reasons for this rather high failure rate include compromised healing at the bone‐tendon junction, as well as degenerative changes in the musculotendinous unit. Therefore, over the last twenty years research has focused on biological augmentation of RC healing, including the application of scaffolds. Although the augmentation of rotator cuff tears using extra‐cellular matrix (ECM) patches, such as porcine small intestine submucosa, has shown improved histological properties at the insertion site [14], no difference in the clinical outcome occurs in the setting of a chronic and large RC tear [28]. Alternatively, using allografts for augmenting RC tears has shown some favourable results [19]. Even though allograft usage is surging in the treatment of large and massive RC tears, there are potential risks with allogenic ECMs because micro fragments of DNA can still be traced in most tested patches, which can thus induce inflammatory reactions in the host [11]. Among all types of patches used to augment RC tears, autologous tissue has shown the most promising results [29]. Kataoka et al. [16] compared the healing process of rotator cuff repair with fascia lata augmentation and without it. They suggested that the fascia lata autograft could enhance healing in the repaired site. More recently, Sun et al. [26] demonstrated that the implementation of bone‐tendon autografts in RC tears in rats, can restore the native tendon enthesis.

Biomechanical evaluation demonstrated that ultimate load‐to‐failure of the Graft group gradually increased with time, and specifically it was significantly higher than that of the Defect group at 12 weeks after repair. Moreover, at the same time‐point the Graft group exhibited greater stiffness than that of the Defect group. Naturally, the normal reference shoulders showed greater tensile strength than the Graft group at 12 weeks, which in turn was greater than the Graft group at 6 weeks after repair. Additionally, the modes of failure were consistent with the biomechanical results. Αt 12 weeks after repair only one specimen of the Graft group failed at the footprint, suggesting strong tendon‐to‐bone healing and fixation. Notably, open rotator cuff repair with transosseous fixation has demonstrated good to excellent clinical outcomes in humans [17] and comparable results to anchor repair in a variety of experimental biomechanical studies in both humans and rabbits [2, 7]. Our findings show that the extra‐synovial autograft improves the biomechanical properties of the subscapularis tendon in rabbits when used to repair large and chronic tears. Histological examination revealed a robust wound‐healing process following transection and repair of the graft, indicative of extra‐synovial tendons healing [1]. Moreover, the tendinous stamp of the grafts finally anchored to the humeral head in all specimens, forming a fibrocartilage like structure.

This study has a number of limitations that have to be acknowledged. First and foremost, this research was based on creating RC tears on an animal model and thus the results cannot fully reflect human RC disease. However, the rabbit subscapularis model is considered the most appropriate experimental model for this purpose [11]. Second, we speculated that the healing of RC in rabbits is analogous to the human RC, but the vascularity, growth factors and even the time course of healing process may differ from humans in many unknown ways to us. Interestingly, the pace of cuff degeneration in rabbits varies from that observed in humans. Previous studies in the rabbit supraspinatus muscle have shown fatty infiltration beginning as early as 4 weeks, with a peak at 6 weeks and with slow reversal by 12 weeks after a tear [3]. Moreover, histological studies have demonstrated that the rabbit subscapularis muscle undergoes chronic changes, such as fatty infiltration and muscle atrophy, at 6 weeks after a tear, with a rise of up to 11% in fat content and a decrease of up to 19% in muscle belly [23]. Therefore, the entire authorship team agreed that the 6 week interval is a reasonable amount of time to create a chronic tear model. Third, we only evaluated histologically the tendon‐to‐bone junction without the subscapularis muscle. We did not collect any information regarding muscle atrophy and fatty infiltration mainly because our study was focused on tendon healing. In spite of those limitations, our study has also several strengths. In contrast to most other experimental studies where acute tears have been investigated, we used a chronic tear model which allows tendon retraction, fatty infiltration and muscle atrophy to occur, leading to more reliable results for rotator cuff disease. Furthermore, two time‐points after repair (6 and 12 weeks) were studied, giving us more information about how the healing process evolves while the recovery continues over a 3‐month period after repair.

## CONCLUSIONS

According to our results, in vivo data support the efficacy of extra‐synovial autograft interposition in repairing chronic and large RC tears in a rabbit SSC model. Extra‐synovial autografts were capable of enhancing the biomechanical properties of the repaired tendons, as evidenced by increased tensile strength, and forming new connective tissue simulating a fibrocartilage zone, as revealed by histological evaluation.

## AUTHOR CONTRIBUTIONS

All authors contributed to the study conception and design. Material preparation and data collection were performed by Iosafat Pinto and Lazaros Kostretzis. Data analysis was performed by Iosafat Pinto, Lazaros Kostretzis, Konstantinos Katakalos, George Kazakos, Angeliki Cheva, Athanasios Chatzisotiriou, Pericles Papadopoulos and Konstantinos Ditsios. The first draft of the manuscript was written by Iosafat Pinto and all authors commented on previous versions of the manuscript. All authors read and approved the final manuscript.

## CONFLICT OF INTEREST STATEMENT

The authors declare no conflict of interest.

## ETHICS STATEMENT

Approval was obtained from the Bioethics Committee of Aristotle University of Thessaloniki (Approval Number: 4108) and from the Animal Health Department of Regional Directorate of Central Macedonia (Approval Number: 449339‐2585). Informed consent was not applicable to this study.

## Data Availability

The data sets used and analyzed during the current study are available from the corresponding author on reasonable request.
